# Smac mimetic induces cell death in a large proportion of primary acute myeloid leukemia samples, which correlates with defined molecular markers

**DOI:** 10.18632/oncotarget.10390

**Published:** 2016-07-02

**Authors:** Sonja C. Lueck, Annika C. Russ, Ursula Botzenhardt, Richard F. Schlenk, Kerry Zobel, Kurt Deshayes, Domagoj Vucic, Hartmut Döhner, Konstanze Döhner, Simone Fulda, Lars Bullinger

**Affiliations:** ^1^ Department of Internal Medicine III, University Hospital Ulm, Ulm, Germany; ^2^ Early Discovery Biochemistry, Genentech, Inc., South San Francisco, CA, USA; ^3^ Institute for Experimental Cancer Research in Pediatrics, Goethe-University, Germany; ^4^ German Cancer Consortium (DKTK), Heidelberg, Germany; ^5^ German Cancer Research Center (DKFZ), Heidelberg, Germany

**Keywords:** smac mimetic, IAP proteins, apoptosis, acute myeloid leukemia (AML), gene expression profiling (GEP)

## Abstract

Apoptosis is deregulated in most, if not all, cancers, including hematological malignancies. Smac mimetics that antagonize Inhibitor of Apoptosis (IAP) proteins have so far largely been investigated in acute myeloid leukemia (AML) cell lines; however, little is yet known on the therapeutic potential of Smac mimetics in primary AML samples. In this study, we therefore investigated the antileukemic activity of the Smac mimetic BV6 in diagnostic samples of 67 adult AML patients and correlated the response to clinical, cytogenetic and molecular markers and gene expression profiles. Treatment with cytarabine (ara-C) was used as a standard chemotherapeutic agent. Interestingly, about half (51%) of primary AML samples are sensitive to BV6 and 21% intermediate responsive, while 28% are resistant. Notably, 69% of ara-C-resistant samples show a good to fair response to BV6. Furthermore, combination treatment with ara-C and BV6 exerts additive effects in most samples. Whole-genome gene expression profiling identifies cell death, TNFR1 and NF-κB signaling among the top pathways that are activated by BV6 in BV6-sensitive, but not in BV6-resistant cases. Furthermore, sensitivity of primary AML blasts to BV6 correlates with significantly elevated expression levels of *TNF* and lower levels of *XIAP* in diagnostic samples, as well as with *NPM1* mutation. In a large set of primary AML samples, these data provide novel insights into factors regulating Smac mimetic response in AML and have important implications for the development of Smac mimetic-based therapies and related diagnostics in AML.

## INTRODUCTION

Acute myeloid leukemia (AML) is a genetically heterogeneous disease with a multi-step pathogenesis [1]. Leukemogenesis is considered to require deregulation of at least two different cellular processes that lead to (i) enhancement of proliferation and survival and (ii) impairment of differentiation [2]. In recent years, many chromosomal aberrations have been identified, which alter normal gene function or expression, thereby contributing to leukemic transformation. Furthermore, many of these cytogenetic aberrations provide important diagnostic and prognostic information [3]. In the large group of cytogenetically normal AML (CN-AML, 40–50% of all AML cases), which show no chromosomal aberrations in conventional banding analysis, currently the identification of novel gene mutations allows dissection of CN-AML into prognostic subgroups [4], and mutations affecting *CEBPA* and *NPM1* are considered as provisional AML entities in the WHO classification [5].

As the hematological compartment is characterized by a fast turnover of cells, a tight regulation of cell survival and cell death is of special importance [6]. Therefore, too little cell death can contribute to a proliferative advantage of transformed cells. Apoptosis is one of the best characterized forms of programmed cell death, which is typically deregulated in most, if not all, cancers [7]. Apoptosis is engaged via ligation of death receptors at the cell surface (extrinsic pathway) or via mitochondria (intrinsic pathway) [8]. Since most current chemotherapeutic strategies depend on intact cell death signaling within cancer cells for their cytotoxic effects, deregulation of cell death programs can lead to treatment resistance [9].

Inhibitors of Apoptosis (IAP) proteins, a family of antiapoptotic proteins comprising e.g. x-linked IAP (XIAP), cellular IAP (cIAP)1 and cIAP2, are known to play a crucial role in many types of human cancer [10]. Also in leukemia, IAP proteins have been associated with chemoresistance, disease progression and poor prognosis [11]. Therefore, IAP proteins are considered as relevant targets for therapeutic intervention and several small-molecule inhibitors have been designed to neutralize IAP proteins [10]. For example, second mitochondria-derived activator of caspases (Smac) mimetics mimick the mitochondrial intermembrane space protein Smac, an endogenous antagonist of IAP proteins that is released into the cytosol during apoptosis [10]. Currently, several Smac mimetics are being tested in clinical trials [12]. We previously demonstrated in acute lymphoblastic leukemia (ALL) and chronic lymphocytic leukemia (CLL) that small-molecule antagonists of IAP proteins can sensitize cells for Tumor-Necrosis-Factor-related apoptosis-inducing ligand (TRAIL)-, CD95- or chemotherapy-induced apoptosis [13–16]. In AML, we recently reported that Smac mimetics can prime cells for several cytotoxic agents that are being used in current treatment protocols, i.e. ara-C and epigenetic drugs such as demethylating agents and histone deacetylase inhibitors (HDACIs) [17–19].

However, these previous studies on Smac mimetics in AML largely embark on AML cell lines and little is yet known about the response of primary AML samples towards treatment with Smac mimetics. In this study, we therefore investigated whether or not primary AML samples are sensitive to the Smac mimetic BV6 that antagonizes XIAP, cIAP1 and cIAP2 [20], and if so, which molecular, cytogenetic or clinical markers correlate with treatment response.

## RESULTS

### Primary AML samples show a differential response to the standard chemotherapeutic drug ara-C and to the Smac mimetic BV6

**Figure 1:**
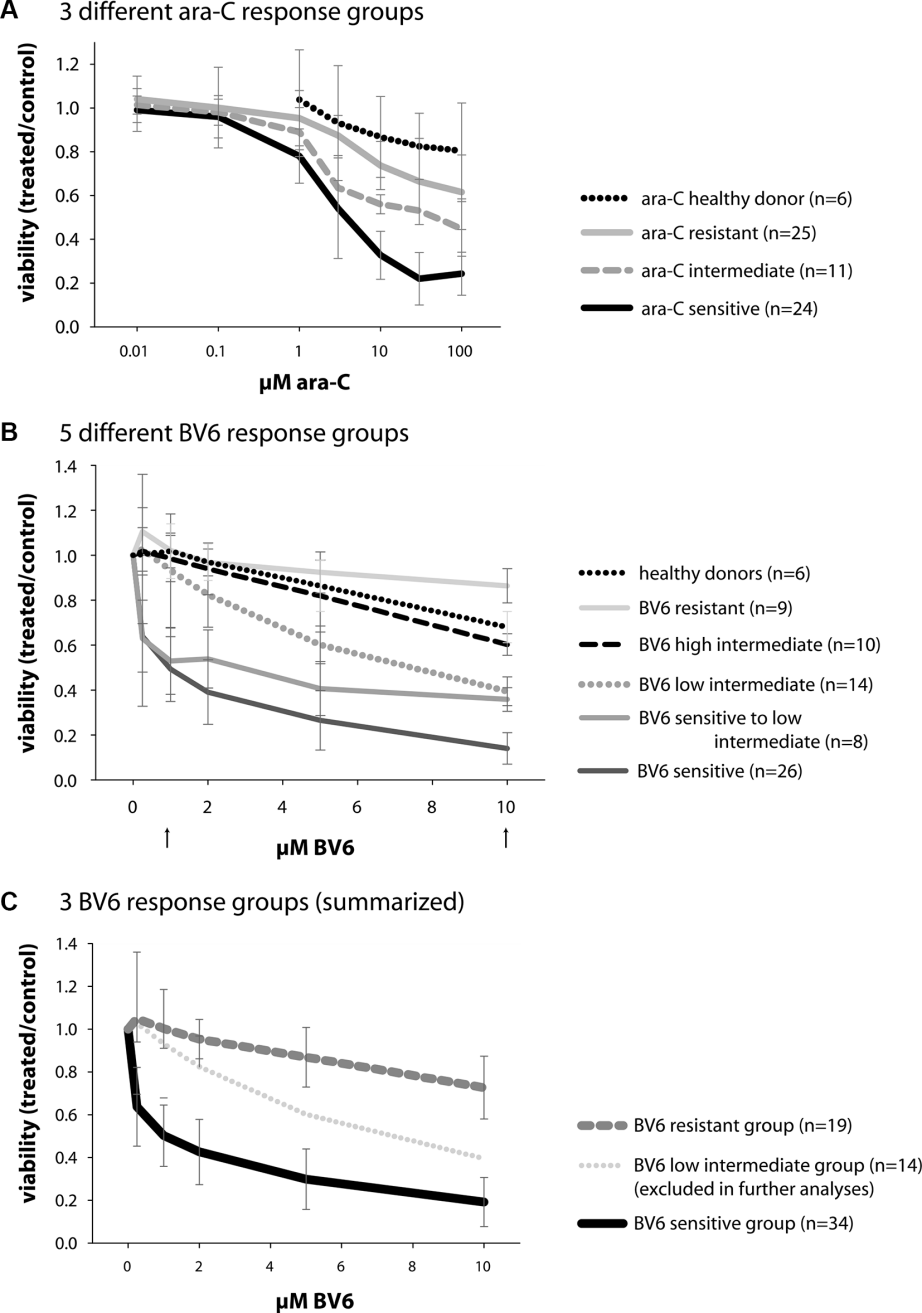
Primary AML sample viability after 24 hours of treatment with either BV6 or ara-C (**A**) 60 primary AML samples and 6 healthy donor samples, treated with ara-C; mean and SD of the three different ara-C response groups are shown (color code independent of BV6 response), which were labeled sensitive (*n* = 24), intermediate, i.e. moderate response (*n* = 11), and resistant (*n* = 25). The healthy donor samples were classified as resistant to ara-C treatment. (**B**) 67 primary AML samples and 6 healthy donor samples, treated with BV6; mean and SD of the five different BV6 response groups defined by their respective viability at 1 and 10 μM BV6 (indicated by arrows) are shown; 26 samples were labeled BV6-sensitive, 8 were sensitive to low intermediate, 14 were low intermediate, 10 were high intermediate, and 9 samples were labeled BV6-resistant. The healthy donors were classified as high intermediate to resistant. (**C**) Classification of 67 AML samples into 3 different BV6 response groups, according to their individual sensitivity: sensitive (*n* = 26 + 8 = 34), low intermediate (*n* = 14, later excluded in further analyses), and resistant (*n* = 10 + 9 = 19) cases.

To explore the therapeutic potential of Smac mimetics in primary AML samples, we investigated the *in vitro* sensitivity to the preclinical Smac mimetic BV6 that antagonize XIAP, cIAP1 and cIAP2 [20] in a large set of 67 newly diagnosed AML patients. To this end, we treated mononuclear cells (mostly leukemic blasts) derived from AML patients at diagnosis for 24 hours with BV6 or with ara-C, which served as a standard chemotherapy control, and determined cell viability (Figure 1). Treatment response to ara-C ranged from mean EC_50_ values of 3.9 μM (ara-C sensitive group) to 50 μM (ara-C intermediate, i.e. moderate response group) and > 100 μM (ara-C resistant group), with a total range of 1.2 μM to > 100 μM (Figure 1A). By comparison, mononuclear cells of healthy donors remained largely resistant towards ara-C (Figure 1A).

Treatment with BV6 revealed distinct response groups, which were defined by their respective cell viability at 1 μM BV6 and 10 μM BV6 (Figure 1B, arrows). Samples with less than 75% viability at 1 μM BV6 and less than 25% viability at 10 μM BV6 were defined as BV6-”sensitive” (*n* = 26). Those with less than 75% viability at 1 μM BV6 and 25–50% viability at 10 μM BV6 were defined as BV6-”sensitive to low intermediate” (*n* = 8). All other response groups included samples with more than 75% viability at 1 μM BV6, which were further subdivided by their viability at 10 μM BV6 into BV6 “low intermediate” (*n* = 14) with 25–50% viability, BV6 “high intermediate” (*n* = 10) with 50–75% viability and BV6 “resistant” (*n* = 9) with more than 75% viability at 10 μM BV6. These five BV6 response subgroups were then compiled into three BV6 response groups, i.e. BV6-sensitive (*n* = 34/67; 51%) comprising “sensitive” and “sensitive to low intermediate” samples, BV6 “low intermediate” (*n* = 14/67; 21%) and BV6-resistant (*n* = 19/67; 28%) containing both “high intermediate” and “resistant” samples (Figure 1C). Thus, about half of the primary AML samples proved to be sensitive to treatment with BV6. Of note, about 42% of ara-C-resistant samples were BV6-sensitive and another 27% fell into the BV6 low intermediate response group, adding up to 69% of ara-C-resistant samples with a good to fair response to BV6. The group “low intermediate” was excluded from further analyses to obtain a clear differentiation of BV6-sensitive and -resistant characteristics.

### Combination treatment of BV6 and ara-C

**Figure 2:**
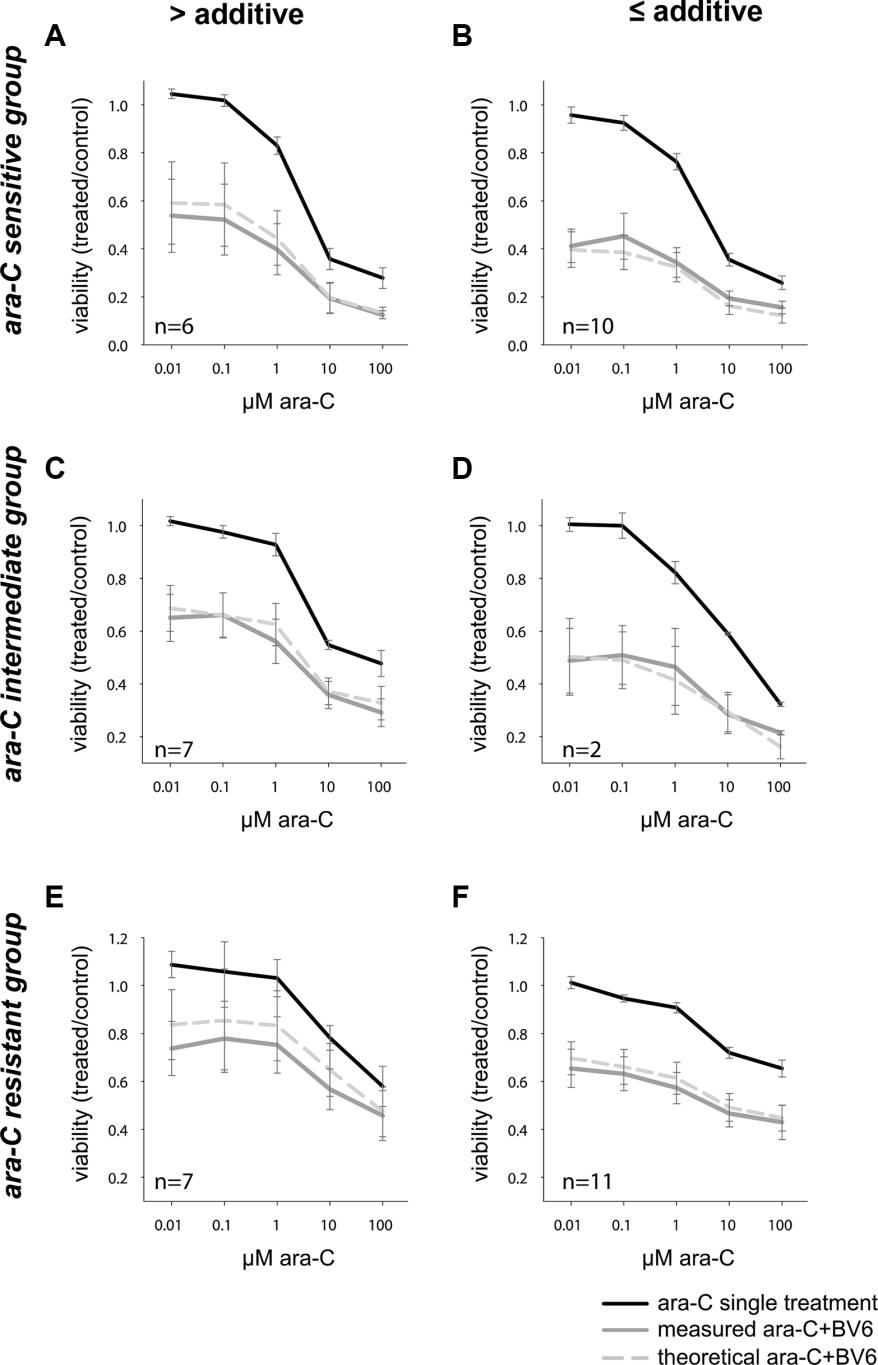
Viability after 24 hours of combination treatment of ara-C and IAP inhibitor BV6 in primary AML samples Combination of ara-C (increasing doses as indicated) with BV6 (single dose: 2 μM), according to the respective ara-C response group (sensitive (**A**, **B**), intermediate (**C**, **D**), resistant (**E**, **F**), see also Figure 1) and the achieved effect of the combination (more than additive (A, C, E) or additive and less than additive (B, D, F)). Mean and SD of all samples in the respective group (sample size as indicated in each graph) are shown, with the three curves representing ara-C single treatment, combination of ara-C with a constant dose of 2 μM BV6 (measured ara-C+BV6), and the theoretical viability of this combination, as defined by the Bliss independence rule (theoretical ara-C + BV6).

As experimental therapies might be more effective in combination with standard chemotherapy, we next assessed the potential of BV6 to sensitize AML cells for ara-C-induced apoptosis. To this end, we treated primary AML samples simultaneously with a low concentration of BV6 (2 μM) and increasing concentrations of ara-C for 24 hours. In general, we observed an additive or more than an additive effect of both drugs, as the measured cell viability after combination treatment was at or below the cell viability predicted for an additive effect according to Bliss' independence rule in most conditions [21, 22] (Figure 2).

### Sensitivity of primary AML samples to BV6 correlates with molecular markers

To find out which AML subgroup might benefit from treatment with Smac mimetics such as BV6, we correlated clinical, cytogenetic and molecular genetic markers with BV6 sensitivity (Table 1). While there was no correlation with age, sex and cytogenetics, there was a trend detectable for internal tandem duplication of the *FLT3* gene (*FLT3*-ITDs) being more prevalent in the BV6-sensitive group (Table 1, 45% versus 27%; *P* =.1141). Of note, *NPM1* mutations were significantly more prevalent in BV6-sensitive compared to BV6-resistant cases (Table 1, 58% versus 20%; *P* = .0076). There was no significant difference in the proportion of *NPM1*-mutated cases without *FLT3*-ITD to *NPM1*-mutated cases with *FLT3*-ITD between BV6-sensitive and -resistant cases (data not shown). Additionally, we found a significant association between *CEBPA* mutations and BV6 resistance (Table 1, 22% versus 0%; *P* = .0144).

**Table 1:** Correlation of BV6 treatment sensitivity with clinical, cytogenetic and molecular marker distribution

	BV6-sensitive	BV6-resistant	*P*-value	
	*n* = 34	*n* = 19	Pearson chi^2^	Mann-Whitney *U*
**Age**	52 (21–77)	57 (20–76)		0.9484
**Sex:**				
**Male**	15/31	11/18	0.3896	
**Female**	6/31	7/18		
**Karyotype:**				
**normal**	18/30	7/16	0.2920	
**CBF**	5/30	3/16	0.8591	
**t(15;17)**	1/30	0/15	0.4745	
**complex**	1/30	2/16	0.2304	
**other**	6/30	4/16	0.6954	
**Molecular markers:**				
***FLT3* ITD MUT**	14/31	4/15	0.1141	
***FLT3* TKD MUT**	4/31	0/15	0.1454	
***CEBPA* MUT**	0/20	2/9	0.0144*	
***NPM1* MUT**	18/31	3/15	0.0076**	

Data on clinical, cytogenetic and molecular markers were available for 31/34 BV6-sensitive and 18/19 BV6-resistant cases.

### GEP in diagnostic samples of BV6-sensitive versus -resistant cases suggests marker genes to predict response

The identification of molecular markers to select patients, which likely respond to a given treatment, is crucial for any novel therapeutic strategy. In this respect, GEP represents a valuable approach, as it allows an unbiased screen of a large number of candidate genes. Therefore, we profiled gene expression of six BV6-sensitive AML samples, and compared the expression pattern to the profiles of six BV6-resistant AML samples. GEP was done in diagnostic (untreated) material, and samples of each group were matched with regard to age, sex, and karyotypes, as far as possible (Supplementary Table 2). Class comparison analysis revealed 25 genes to be differentially expressed between BV6-sensitive and -resistant samples (Table 2, *P* < .01). These included several candidate tumor suppressor genes, such as *ABLIM1*, *CYFIP2*, *VPS13A*, *SERPINI1*, *TET2*, *HTRA4*, *UBE2L6*, as well as oncogenes/genes implicated as biomarkers in other cancers, such as *FAM65B* and *TCF7L2* (Table 2).

**Table 2:** Class comparison (CC) results for BV6-resistant (*n* = 6) vs. -sensitive cases *n* = 6)

no. in CC	Parametric *p*-value	Geom mean of intensities in BV6-resistant samples	Geom mean of intensities in BV6-sensitive samples	Fold-change	Probe set	Gene symbol
1	0.0001	97.17	44.73	2.17	200965_s_at	*ABLIM1*
2	0.0005	7.25	4.77	1.52	1557167_at	*HCG11*
3	0.0009	70.64	22.96	3.08	220999_s_at	*CYFIP2*
4	0.0017	70.31	38.83	1.81	243601_at	*LOC285957*
5	0.0021	61.81	28.73	2.15	227988_s_at	*VPS13A*
6	0.0027	23.63	12.47	1.89	205352_at	*SERPINI1*
7	0.0029	28.49	55.48	0.51	240451_at	NA
8	0.0034	85.26	143.04	0.60	238851_at	*ANKRD13A*
9	0.0035	98.26	257.72	0.38	239167_at	NA
10	0.0040	29.68	52.47	0.57	235461_at	*TET2*
11	0.0044	32.45	12.59	2.58	1568658_at	*C2orf74*
12	0.0047	159.32	264.85	0.60	1560926_at	NA
13	0.0047	12.51	20.54	0.61	1553706_at	*HTRA4*
14	0.0052	26.49	52.54	0.50	244550_at	NA
15	0.0055	30.29	15.15	2.00	227987_at	*VPS13A*
16	0.0070	489.19	249.39	1.96	201649_at	*UBE2L6*
17	0.0072	169.82	53.91	3.15	209829_at	*FAM65B*
18	0.0078	17.35	28.15	0.62	237548_at	NA
19	0.0082	216.14	401.72	0.54	244753_at	NA
20	0.0082	632.50	325.33	1.94	212761_at	*TCF7L2*
21	0.0082	89.75	45.69	1.96	202125_s_at	*TRAK2*
22	0.0084	63.47	29.83	2.13	215694_at	*SPATA5L1*
23	0.0094	14.88	25.49	0.58	232405_at	NA
24	0.0094	41.44	22.31	1.86	229986_at	*ZNF717*
25	0.0095	330.43	509.22	0.65	213742_at	*SFRS11*

For some probe sets, no gene symbol is annotated to date (NA).

### BV6 activates several cell death-related pathways in BV6-sensitive, but not in -resistant cases

To gain unbiased insights into BV6-stimulated signaling pathways in primary AML patient samples we performed whole-genome GEP of BV6-sensitive and -resistant samples after 24-hour-treatment with either BV6 or DMSO (Supplementary Table 3). Interestingly, pathway comparison analysis revealed a significant enrichment of a BV6-response signature for cell death-related pathways in BV6-sensitive samples, such as *Apoptotic DNA fragmentation and tissue homeostasis*, *TNFR1 Signaling Pathway*, *D4-GDI Signaling Pathway, Caspase Cascade in Apoptosis*, as well as *Opposing roles of AIF in Apoptosis and Cell Survival* and *NF-κB Signaling Pathway* (Supplementary Table 4). In total, 12 Biocarta pathways were significantly differentially regulated between BV6- and DMSO-treated samples in BV6-sensitive cases (Table 3, *P* < .05, Least Squares (LS)/ Kolmogorov-Smirnov (KS) test). In contrast, GEP of BV6-resistant cases revealed no differential regulation of these cell death-related pathways in our cohort. Here, *CTL-mediated immune response against target cells* was found to be most significantly enriched within the differentially regulated genes, notably with a lower expression of the key player *FAS* in BV6-treated cells (Supplementary Table 4). Furthermore, *PTEN-dependent cell cycle arrest and apoptosis* was also listed to differ between BV6- and DMSO-treated BV6-resistant samples, with a lower expression of *PTEN* itself in BV6-treated samples (Supplementary Table 4). Overall, 26 Biocarta pathways were found to have more members significantly differentially expressed among BV6- and DMSO-treated samples in BV6-resistant cases (Supplementary Table 4, *P* < .05, LS/KS test).

**Table 3:** Selected Biocarta pathways which were significantly differentially expressed between BV6- and DMSO-treated samples (BV6-sensitive cases)

Biocarta Pathway	Pathway description	# of genes	LS *p*-value	KS *p*-value	Efron-Tibshirani's *p*-value
h DNAfragment Pathway	Apoptotic DNA fragmentation and tissue homeostasis	15	0.00003	0.04441	< 0.005
h smP athway	Spliceosomal Assembly	22	0.00003	0.04504	< 0.005
h il18 Pathway	IL 18 Signaling Pathway	7	0.00136	0.00337	< 0.005
h rab Pathway	Rab GTPases Mark Targets In The Endocytotic Machinery	25	0.00242	0.04861	< 0.005
h tnfr1 Pathway	TNFR1 Signaling Pathway	37	0.0038	0.00918	< 0.005
h cpsf Pathway	Polyadenylation of mRNA	9	0.00931	0.01273	< 0.005
h eif2 Pathway	Regulation of eIF2	18	0.01638	0.01819	< 0.005
h d4gdi Pathway	D4-GDI Signaling Pathway	21	0.02449	0.28193	< 0.005
h RacCycD Pathway	Influence of Ras and Rho proteins on G1 to S Transition	34	0.02801	0.05138	< 0.005
h prc2 Pathway	The PRC2 Complex Sets Long-term Gene Silencing Through Modification of Histone Tails	13	0.03252	0.10773	< 0.005
h bard1 Pathway	BRCA1-dependent Ub-ligase activity	5	0.04497	0.08486	< 0.005
h caspase Pathway	Caspase Cascade in Apoptosis	36	0.04637	0.51603	< 0.005
h aif Pathway	Opposing roles of AIF in Apoptosis and Cell Survival	5	0.29515	0.46769	< 0.005
h NF-κB Pathway	NF-κB Signaling Pathway	25	0.42002	0.08742	< 0.005

In total, 60 Biocarta pathways were differentially regulated specifically in BV6-sensitive cases (BV6 vs. DMSO treatment). Here we show an excerpt of 12 Biocarta pathways (*P* <.05 in LS/KS test), plus two others that were especially relevant.

### Gene expression levels of selected cell death-related genes correlate with BV6 sensitivity

**Figure 3:**
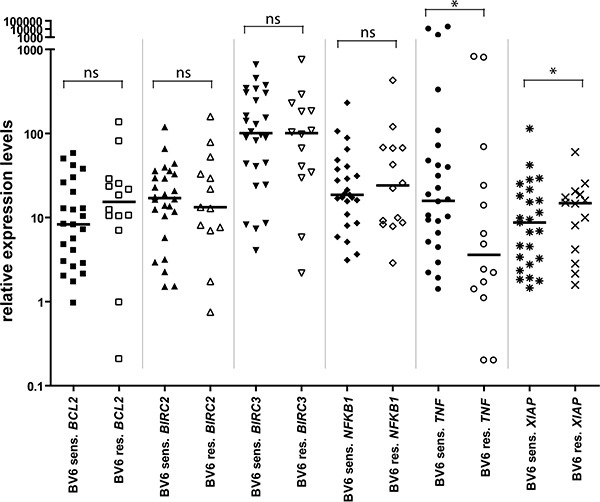
Comparison of gene expression levels of several apoptosis-relevant genes in BV6-sensitive (*n* = 26) vs. -resistant (*n* = 14) cases, measured by qRT-PCR in 40 primary AML samples All expression levels were normalized to *ACTB* expression levels. Shown are individual expression values as scatter plot, with closed symbols for BV6-sensitive and open symbols for BV6-resistant samples, as well as group median and statistical test results (Mann-Whitney *U* test; *P < 0.05; ns = non-significant, *P* ≥ 0.05).

Based on these GEP studies, we further investigated whether the *in vitro* response of primary AML samples to BV6 correlates with expression levels of a defined set of cell death-related genes. To this end, we measured expression levels of *BIRC2* (encoding cIAP1), *BIRC3* (encoding cIAP2), *XIAP*, *TNF*, *NF-κB1* and *BCL2* in selected representative untreated diagnostic samples by qRT-PCR (Figure 3). Interestingly, expression levels of *XIAP* were significantly lower in BV6-sensitive cases compared to BV6-resistant cases (Figure 3, *P* = .0241). In addition, BV6-sensitive samples exhibited significantly higher expression levels of *TNF* than BV6-resistant samples (Figure 3, *P* = .0493). No significant difference was found for *BCL2*, *NF-κB1* and *BIRC2* expression between BV6-sensitive and -resistant cases (Figure 3). Thus, constitutively low expression of *XIAP* and constitutively high levels of *TNF* correlate with the sensitivity of primary AML samples towards treatment with BV6.

## DISCUSSION

IAP proteins represent relevant targets for therapeutic intervention in AML, since they are expressed at high levels and contribute to evasion of cell death [11]. In the present study, we report that about half of the 67 investigated primary AML samples at diagnosis are sensitive to the Smac mimetic BV6 including about two thirds of ara-C-resistant cases, while mononuclear cells from healthy donors remain largely unaffected. In addition, BV6 enhances the antileukemic activity of the standard chemotherapeutic drug ara-C in an additive manner. Our present study is the largest evaluation so far of Smac mimetic's antileukemic activity in primary, newly diagnosed AML samples, thus highlighting its clinical relevance. By comparison, most previous studies focused on AML cell lines included only few, if any, primary samples [17–19, 23–29]. We previously reported that inhibition of IAP proteins by small-molecule inhibitors does not sensitize unstimulated or phytohemoagglutinin-activated peripheral blood lymphocytes for ara-C-induced cell death [16]. Also, we demonstrated that treatment with Smac mimetic has no adverse effect on colony formation of normal human CD34^+^ hematopoietic cells freshly isolated from healthy human donors at concentrations that are cytotoxic against leukemia cells [30]. However, cytokine release syndrome has been reported as a dose-limiting toxicity in a recent phase I study [31].

Using whole-genome GEP, we identify a number of functional categories related to cell death that are significantly induced by BV6 treatment in sensitive samples only. Several categories are linked to apoptosis, consistent with the role of IAP proteins in the regulation of programmed cell death. These findings are in line with activation of cell death in sensitive AML samples via IAP inhibition by Smac mimetic. In addition, our GEP studies identify *NF-κB Signaling Pathway* as another pathway that is activated by BV6 treatment in sensitive samples. This is in line with the reported role of IAP proteins in NF-κB signaling. cIAP1 and cIAP2 are positive regulators of canonical NF-κB signaling and suppress the non-canonical NF-κB pathway [20, 32–34]. Accordingly, depletion of cIAP proteins by Smac mimetics such as BV6 has been shown to stimulate non-canonical NF-κB signaling via accumulation of NF-κB-inducing kinase (NIK) [20, 34], a critical kinase upstream in the non-canonical NF-κB pathway that is constitutively degraded by cIAP1 and cIAP2. This leads to upregulation of NF-κB target genes such as TNFα, which engages an autocrine/paracrine loop by binding to its cognate receptor TNFR1 on the cell surface to trigger cell death in the presence of Smac mimetics [20, 34, 35]. Consistently, *TNFR1* *Signaling Pathway* is identified as another signaling cascade in BV6-treated responsive AML samples. Also, NF-κB signaling has previously been implicated in mediating Smac mimetic-induced cell death [36, 37]. Of note, we recently identified by GEP cell death and NF-κB among the top pathways regulated by BV6 in responsive primary CLL samples as well as in primary core-binding factor (CBF) AML samples [38], underscoring the general relevance of these signaling pathways for Smac mimetic-mediated antitumor activity. While that study also revealed redox signaling as another pathway in BV6-sensitive primary CLL samples as well as in primary CBF AML samples [38], we did not find redox-related signaling cascades among the top regulated pathways in the present set of AML samples, pointing also to some differences among these types of leukemia.

Furthermore, in the present study we identify low constitutive levels of *XIAP* and high constitutive expression of *TNF* as parameters that correlate with sensitivity to BV6 in AML samples. Since low XIAP expression favors the induction of cell death, this finding is in line with the observed response to Smac mimetics. It is interesting to note that constitutive TNFα production has previously been linked to sensitivity of human cancer cell lines towards Smac mimetics [39]. Also, TNFα/TNFR1 autocrine/paracrine signaling has been shown to trigger cell death in the presence of Smac mimetics that facilitate TNFα-mediated cell death by depleting cIAP proteins [20, 34, 39]. We previously reported that the TNFα-blocking antibody Enbrel significantly reduces BV6/ara-C-induced cell death in AML cell lines [17], consistent with an autocrine/paracrine TNFα loop mediating cell death. However, there is also evidence showing that Smac mimetics can trigger cell death via death receptor 5 independently of TNFα/TNFR1 signaling [36].

Moreover, by whole-genome GEP we identify a set of 25 genes that are differentially expressed among untreated BV6-sensitive and -resistant cases, which might serve as biomarkers to identify BV6 sensitivity prior to treatment. In this regard, *SERPINI1* is of special interest, as it was found to be a biomarker for hepatocellular carcinoma [40], and expression of *SERPINI1* has been shown to be regulated by c-Myc [41]. Furthermore, *TET2* and *HTRA4* were expressed at lower levels in BV6-resistant samples and might also be at least partly involved in treatment resistance. *TET2* has been implicated in leukemia and associated with decreased overall survival in AML, as its loss of function was found to impair differentiation and favor myeloid tumorigenesis [42–45]. *HTRA4* is thought to be involved in the modulation of apoptosis and chemotherapy-induced cytotoxicity with a tumor-suppressive role [46]. In contrast to *TET2* and *HTRA4*, the transcription factor *TCF7L2* was less expressed in BV6-sensitive samples. *TCF7L2* has been implicated in AML [47, 48], regulates *MYC* expression and has an influence on survival and proliferation [49–51]. While the predictive power of these gene expression differences for BV6 treatment sensitivity requires further validation, the candidates revealed by our analysis provide a starting point for subsequent studies.

Of the tested molecular markers, we identify *CEBPA* and *NPM1* mutations to be differentially distributed among BV6-sensitive and -resistant AML samples. Here, *NPM1* mutations are more prevalent and *CEBPA* mutations less prevalent in BV6-sensitive cases. As far as *NPM1* mutations are concerned, the association with BV6 sensitivity proved to be significant even when taking into account *FLT3*-ITD. While these data indicated that *NPM1*-mutated AML cases might be an AML subgroup that responds to BV6 treatment, additional markers may be necessary to prospectively define sensitive AML cases, as *IDH* mutations have been reported to confer adverse prognosis in AML with *NPM1* mutations [52]. Beyond *CEBPA* and *NPM1* mutations, our previous work in CBF AML identified a higher sensitivity to the Smac mimetic BV6 in the subgroup with superior outcome [23].

As several Smac mimetics are currently being tested in early clinical trials [12], the identification of molecular markers to select patients which likely respond to Smac mimetics becomes more and more important. Molecular markers identified by GEP or by genomic analysis represent valuable approaches in this respect. Our results point towards Smac mimetics as a novel therapeutic option in AML, especially in patients with *NPM1* mutations, low XIAP expression or high TNF expression. These findings are expected to have important implications for the design of Smac mimetic-based protocols in the treatment of AML.

## MATERIALS AND METHODS

### Primary AML patient samples

Samples [total *n* = 67, *n* = 24 peripheral blood (PB) and *n* = 43 bone marrow (BM) specimens] from adult AML patients at diagnosis were provided by the German-Austrian AML Study Group (AMLSG) with patient-informed consent obtained in accordance with the Declaration of Helsinki and institutional review board approval from all participating centers. Mononuclear cells were Ficoll gradient purified, and the percentage of leukemic cells/blasts was at least 80% following enrichment. Patient age at the time of diagnosis ranged from 20.3 to 81.7 years (median 57.0 years). Clinical characteristics at the time of diagnosis are detailed in Supplementary Table 1.

### Cell culture and *in vitro* treatment

Primary AML samples were cultivated using RPMI 1640 (Biochrom AG, Berlin, Germany), supplemented with 20% fetal calf serum (FCS) (Sigma-Aldrich, St. Louis, MO, USA), 2 mM L-Glutamin (Biochrom AG), and Penicillin/Streptomycin (GIBCO, Invitrogen Corporation, Grand Island, NY, USA). Prior to treatment, cells were stained with trypan blue (Sigma-Aldrich), counted, and diluted to a density of 1.0 × 10^6^ cells/ml. Thawing of viably frozen samples followed the DSMZ (German Collection of microorganisms and cell lines, Braunschweig) guideline. Agents used to treat primary AML samples were DMSO (control/carrier; dimethyl sulfoxide; Sigma-Aldrich), ara-C (cytarabine; cell pharm, Bad Vilbel, Germany), and BV6, a bivalent Smac mimetic that antagonizes XIAP, cIAP1 and cIAP2 [20], was kindly provided by Genentech, Inc. (South San Francisco, CA, USA).

### Viability assays

We performed an ATP-content measurement using the CellTiter-Glo Luminescent Cell Viability Assay (Promega, Madison, WI, USA), which reflects the amount of viable cells per sample. For read-out, we used the GloMax 96 luminometer (Promega GmbH, Mannheim, Germany). Furthermore, we performed flow cytometry in selected samples, which were double-stained using Annexin V-PE (BD Pharmingen, Franklin Lakes, NJ, USA) and 7-AAD (7-Amino-Actinomycin D; BD Pharmingen) according to the manufacturer's protocol, and measured using a FACSCalibur (BD). All treated samples were measured after 24 hours of treatment and normalized to appropriate control-treated samples.

### Quantitative RT-PCRs (qRT-PCRs)

For qRT-PCRs, we isolated RNA from samples with TRIzol reagent (Invitrogen, Life Technologies Corporation, Carlsbad, CA, USA). Reverse transcription was done with SuperScript III First-Strand Synthesis System for RT-PCR (Invitrogen), primed with random hexamers and following the manufacturers' protocol. Quantitative real-time RT-PCRs were done with the Fast SYBR Green Master Mix (Applied Biosystems, Life Technologies Corporation, Carlsbad, CA, USA) using a 7900HT Fast Real-Time PCR System (Applied Biosystems) in the fast mode. Primer (custom oligonucleotides, Invitrogen) sequences were (all 5′ to 3′): *NF-κB1* forward TGGAGTCTGGGAAGGATTTG, reverse CGAAGCTGGACAAACACAGA; *TNF* forward CCCCAGGGACCTCTCTCTAA, reverse CAGCTTGAG GGTTTGCTACA; *BCL2* forward ATGTGTGTGGAGAG CGTCAA, reverse ACAGTTCCACAAAGGCATCC; *XIAP* forward CATTCACTTGAGGAGTGTCTGG, reverse TGAAACTGAACCCCATTCGT; *BIRC3* forward CCAAGTGGTTTCCAAGGTGT, reverse TTTTCATCT CCTGGGCTGTC; *BIRC2* forward CCAAGTGGTTT CCAAGGTGT, reverse ATTGGTGGGTCAGCATTTTC; *ACTB* forward AGAGCTACGAGCTGCCTGAC, reverse AGCACTGTGTTGGCGTACAG.

### Gene expression profiling (GEP)

Using GeneChip Human Genome U133 Plus 2.0 Arrays (Affymetrix, Santa Clara, CA, USA) gene expression was profiled in 24 AML samples [12 untreated, diagnostic samples of BV6-resistant (*n* = 6) and sensitive (*n* = 6) cases; 6 paired 24-hours DMSO/BV6 treated cases (*n* = 12 samples) from BV6-sensitive (*n* = 6) and -resistant (*n* = 6) cases]. CEL files (available at gene expression omnibus, accession GSE46819) were normalized and filtered using BRB Array Tools Version 3.7.2 by applying the JustRMA algorithm and previously reported filtering criteria [53].

### Data analysis

Microarray data were analyzed using BRB Array Tools Version 3.7.2 as previously described [54]. Group-wise comparisons of the distributions of clinical and laboratory variables were performed using Mann-Whitney *U* test, unpaired *t*-test with Welch correction, Fisher's exact test, and Pearson chi-squared test, as appropriate. An effect was considered significant, if the *P* value was 0.05 or less. Data were visualized using either SigmaPlot (Systat Software, San Jose, CA, USA) or GraphPad Prism 4 (GraphPad Software, La Jolla, CA, USA).

## SUPPLEMENTARY MATERIALS FIGURES AND TABLES




