# Supplementary material for: Smac mimetic induces cell death in a large proportion of primary acute myeloid leukemia samples, which correlates with defined molecular markers
Source: Oncotarget. 2016 Jul 2;7(31):49539–51. doi: 10.18632/oncotarget.10390 (PMC5226527; doi:10.18632/oncotarget.10390)
Supplement: Supplementary file 1 [file oncotarget-07-49539-s001.pdf]

Smac mimetic induces cell death in a large proportion of primary acute myeloid leukemia samples, which correlates with defined molecular markers

Supplementary Materials

Supplementary Table S1: Clinical characteristics for newly diagnosed AML patients (*n* = 67). See Supplementary_Table_S1

Supplementary Table S2: Clinical, cytogenetic and molecular genetic characteristics of primary AML samples (*n* = 12) profiled on microarrays to obtain BV6 response associated signature. See Supplementary_Table_S2

Supplementary Table S3: Top 100 genes differentially expressed in BV6 versus DMSO treated samples, according to Class comparison analysis. See Supplementary_Table_S3

Supplementary Table S4: Differentially regulated Biocarta pathways among BV6- and DMSO-treated primary AML samples, according to LS/KS permutation testing (Pathway comparison). Light grey script indicated pathways not significant in LS/KS testing, but in GSA test. See Supplementary_Table_S4
