# Supplementary material for: Smac mimetic induces cell death in a large proportion of primary acute myeloid leukemia samples, which correlates with defined molecular markers
Source: Oncotarget. 2016 Jul 2;7(31):49539–51. doi: 10.18632/oncotarget.10390 (PMC5226527; doi:10.18632/oncotarget.10390)
Supplement: Supplementary file 2 [file oncotarget-07-49539-s002.pdf]

Supplemental Table 1: Clinical characteristics for newly diagnosed AML patients (n=67).

# AML	age	sex	karyotype	t(8;21)	inv(16)	t(15;17)	t(9;11)	*FLT3* ITD	*FLT3* TKD	*CEBPA*	*NRAS*	*NPM1*
1	32.8	m	CBF	WT	MUT	WT	WT	WT	WT	WT	WT	WT
2	55.2	w	normal	WT	WT	WT	WT	WT	WT	MUT		MUT
3	61.4	m	del12/normal					MUT	WT	WT		MUT
4	56.1	m	normal	WT	WT	WT	WT	WT	MUT	WT		MUT
5	21.0	m	CBF	WT	MUT	WT	WT	WT	WT	WT	WT	WT
6	57.0	m										
7	50.8	m	normal	WT	WT	WT	WT	WT	MUT	WT		MUT
8	46.1	m	normal	WT	WT	WT	WT	MUT	WT	WT		MUT
9	73.6	w	normal	WT	WT	WT	WT	MUT	WT			MUT
10	46.9	w	normal	WT	WT	WT	WT	MUT	WT	WT		WT
11	32.6	m	inv3; -7	WT	WT	WT	WT	WT	WT	WT		WT
12	76.5	m	normal	WT	WT	WT	WT	WT	WT	WT		MUT
13	47.8	w	normal	WT	WT	WT	WT	MUT	WT	WT		MUT
14	55.9	m	normal	WT	WT	WT	WT	WT	WT	MUT		WT
15	57.5	m	del13	WT	WT	WT	WT	MUT	WT	WT		WT
16	23.7	w	normal	WT	WT	WT	WT	WT	MUT	MUT		WT
17	81.7	w	normal	WT	WT	WT	WT	WT	WT			WT
18	58.5	w	+8	WT	WT	WT	WT	MUT	WT	WT		WT
19	48.5	m	normal	WT	WT	WT	WT	WT	WT	WT		WT
20	44.6	w	normal	WT	WT	WT	WT	MUT	WT	WT		MUT
21	66.9	m	complex	WT	WT	WT	WT	WT	WT			WT
22		m										
23	61.0	m	t(2;14)	WT	WT	WT	WT	MUT	WT			WT
24	26.8	w	+21	WT	WT	WT	WT	WT	WT	MUT		WT
25	33.8	m	CBF	WT	MUT	WT	WT	WT	WT	WT		WT
26	53.5	w	normal	WT	WT	WT	WT	WT	MUT	WT		MUT
27	57.0	w	CBF	WT		WT	WT	WT	WT	WT		WT
28	30.8	w	normal	WT	WT	WT	WT	WT	WT	MUT		WT
29	31.6	w	normal	WT	WT	WT	WT	WT	MUT	WT		MUT
30	58.2	m	normal	WT	WT	WT	WT	WT	WT	WT		MUT
31	28.1	w	CBF	WT	MUT	WT	WT	WT	WT	WT		WT
32	62.2	w	normal	WT	WT	WT	WT	MUT	WT			MUT
33	28.8	m	complex	WT	WT	WT	WT	WT	WT	WT		WT
34	66.6	w	normal	WT	WT	WT	WT	MUT	WT			MUT
35	65.8	m	+8 +4	WT	WT	WT	WT	WT	WT			WT
36	41.8	m	normal	WT	WT	WT	WT	MUT	WT	WT		WT
37	20.3	m	+11	WT	WT	WT	WT	WT	WT			WT
38	75.0	w	normal	WT	WT	WT	WT	MUT	WT			MUT
39	49.6	m	normal	WT	WT	WT	WT	WT	MUT	WT		MUT
40	68.2	m	normal	WT	WT	WT	WT	WT	WT			WT
41												
42	39.5	m	normal	WT	WT	WT	WT	MUT	WT	WT		MUT
43	46.5	w	normal	WT	WT	WT	WT	MUT	WT	WT		MUT
44	71.4	w	normal	WT	WT	WT	WT	MUT	WT			WT
45	61.8	w	t(7;15); t(9;22)	WT	WT	WT	WT	WT	WT			WT
46	71.7	m	normal	WT	WT	WT	WT	MUT	WT			MUT
47	58.2	w	t(15;17)	WT	WT	MUT	WT	WT	WT	WT		WT
48	70.2	w	complex	WT	WT	WT	WT	WT	WT			WT
49	46.6	w	CBF	WT	MUT	WT	WT	WT	WT			WT
50	66.8	w	normal	WT	WT	WT	WT	WT	WT			WT
51	52.1	w	CBF	MUT	WT	WT	WT	WT	WT	WT		WT
52	78.0	w	normal	WT	WT	WT	WT	WT	WT			MUT
53	68.3	m	complex	WT	WT	WT	WT	WT	WT			WT
54	47.7	m	normal	WT	WT	WT	WT	WT	WT	WT		MUT
55	50.6	m		WT	WT	WT	WT	WT	WT	WT		MUT
56												
57	68.7	m	normal	WT	WT	WT	WT	MUT	WT			MUT
58	41.2	w	CBF									
59												
60												
61	57.6	m	normal	WT	WT	WT	WT	MUT	WT	WT		MUT
62	69.5	m	normal	WT	WT	WT	WT	WT	WT			WT
63	72.0	w	normal	WT	WT	WT	WT	WT	WT			WT
64	33.0	m	CBF	WT	MUT	WT	WT	WT	WT	WT		WT
65	76.8	w	normal	WT	WT	WT	WT	WT	WT			MUT
66	70.8	w	+13	WT	WT	WT	WT	WT	WT			WT
67	58.7	m	normal	WT	WT	WT	WT	MUT	WT			MUT

Supplemental Table 2: Clinical, cytogenetic and molecular genetic characteristics of primary AML samples (n=12) profiled on microarrays to obtain BV6 response associated signature.

# AML	age	sex	karyotype	t(15;17)	t(9;11)	*FLT3* ITD	*FLT3* TKD	*CEBPA*	*NPM1*	BV6 response group	ara-C response group
3	61	m	del12/normal	n.a.	n.a.	MUT	WT	WT	MUT	sensitive	resistant
18	58	w	+8	WT	WT	MUT	WT	WT	WT	sensitive	sensitive
29	32	w	normal	WT	WT	WT	MUT	WT	MUT	sensitive	sensitive
49	47	w	CBF	WT	WT	WT	WT	n.a.	WT	sensitive	sensitive
54	48	m	normal	WT	WT	WT	WT	WT	MUT	sensitive	sensitive
65	77	w	normal	WT	WT	WT	WT	n.a.	MUT	sensitive	intermediate
2	55	w	normal	WT	WT	WT	WT	MUT	MUT	resistant	n.a.
12	76	m	normal	WT	WT	WT	WT	WT	MUT	resistant	resistant
15	58	m	del13	WT	WT	MUT	WT	WT	WT	resistant	n.a.
24	27	w	+21	WT	WT	WT	WT	MUT	WT	resistant	sensitive
27	57	w	CBF	WT	WT	WT	WT	WT	WT	resistant	resistant
37	20	m	+11	WT	WT	WT	WT	n.a.	WT	resistant	resistant

Supplemental Table 3: Top 100 genes differentially expressed in BV6 versus DMSO treated samples, according to Class comparison analysis.

160 genes are significant at the nominal 0.01 level of the univariate test

BV6 resistant	Parametric p-value	Geometric mean of intensities (class BV6 /class DMSO)	Probe set	Gene symbol
1	0.00006	1.35835	242082 at	MMAB
2	0.00006	0.89769	225224 at	C20orf112
3	0.00012	1.12781	223788 at	NA
4	0.00028	0.86832	224630 at	C2orf30
5	0.00029	0.93213	203415 at	PDCD6
6	0.00031	1.17885	202864 s at	SP100
7	0.00034	1.15106	218775 s at	WWC2
8	0.00034	0.92858	221970 s at	NOL11
9	0.00053	1.20043	209960 at	HGF
10	0.00055	1.47515	1554769 at	ZNF785
11	0.00063	0.97508	211240 x at	CTNND1
12	0.00070	0.87396	203293 s at	LMAN1
13	0.00079	1.12513	1555781 at	PQLC2
14	0.00084	0.77157	201666 at	TIMP1
15	0.00087	1.26557	205248 at	DOPEY2
16	0.00091	1.28720	234536 at	SARDH
17	0.00093	1.20611	217497 at	TYMP
18	0.00108	1.12826	1554240 a at	ITGAL
19	0.00108	1.10991	223227 at	BBS2
20	0.00118	0.83916	220397 at	MDM1
21	0.00118	1.27389	228755 at	GIGYF1
22	0.00120	0.84090	207319 s at	CDC2L5
23	0.00130	1.37717	210230 at	NA
24	0.00130	1.17975	228058 at	LOC124220
25	0.00136	1.28461	235575 at	SDHALP2
26	0.00136	0.82459	218948 at	QRSL1
27	0.00146	1.15921	243404 at	NA
28	0.00151	0.88725	201368 at	ZFP36L2
29	0.00151	1.04924	218120 s at	HMOX2
30	0.00153	0.82487	211801 x at	MFN1
31	0.00163	1.40238	214395 x at	EEF1D
32	0.00165	1.15671	203388 at	ARRB2
33	0.00168	0.85484	209879 at	SELPLG
34	0.00170	0.86164	229297 at	NA
35	0.00192	0.86405	200614 at	CLTC
36	0.00194	1.11624	228592 at	MS4A1
37	0.00197	1.18237	1562062 at	NBPF10
38	0.00204	0.87248	229752 at	IKZF4
39	0.00213	1.41090	1555423 at	SSH2
40	0.00217	0.89888	201067 at	PSMC2
41	0.00217	0.95432	203583 at	UNC50
42	0.00223	0.81714	1552928 s at	MAP3K7IP3
43	0.00223	1.16600	1566785 x at	LOC728806
44	0.00225	0.87132	207861 at	CCL22
45	0.00226	1.12408	65493 at	HEATR6
46	0.00241	1.08976	209913 x at	KIAA0415
47	0.00249	0.78872	200713 s at	MAPRE1
48	0.00257	1.20702	230628 at	EP400
49	0.00259	1.41324	227575 s at	C14orf102
50	0.00271	1.18378	213053 at	KIAA0841
51	0.00277	1.28724	218859 s at	ESF1
52	0.00287	1.31228	1558948 a at	NA
53	0.00294	0.83217	210178 x at	FUSIP1
54	0.00301	0.83672	225845 at	ZBTB44
55	0.00315	1.25122	222252 x at	UBQLN4
56	0.00319	0.89575	203013 at	ECD
57	0.00328	1.21017	218747 s at	TAPBPL
58	0.00329	0.90544	214864 s at	GRHPR
59	0.00334	1.30223	215635 at	NA
60	0.00339	1.11503	205387 s at	CGB
61	0.00341	0.89664	224009 x at	DHRS9
62	0.00348	0.79662	223073 at	HIATL1
63	0.00349	1.19129	240993 at	UPF3B
64	0.00358	0.86145	223207 x at	PHPT1
65	0.00364	1.04535	210031 at	CD247
66	0.00372	0.72714	227364 at	NA
67	0.00377	1.27414	205255 x at	TCF7
68	0.00380	1.11075	209682 at	CBLB
69	0.00389	1.11007	1556389 at	CNPY3
70	0.00402	1.35641	1569578 at	NA
71	0.00402	0.93663	204299 at	FUSIP1
72	0.00404	0.93181	217493 x at	NCR2
73	0.00406	1.08369	225918 at	NA
74	0.00407	1.19779	230945 at	NA
75	0.00412	1.14112	213965 s at	CHD5
76	0.00412	1.36409	221192 x at	MFSD11
77	0.00413	0.91165	212912 at	RPS6KA2
78	0.00422	1.15125	239120 at	NA
79	0.00445	0.86442	217741 s at	ZFAND5
80	0.00447	0.72731	236165 at	MSL3
81	0.00453	1.17096	231941 s at	MUC20
82	0.00468	0.88967	240927 at	NA
83	0.00469	0.79340	227139 s at	HPS3
84	0.00470	0.80571	217825 s at	UBE2J1
85	0.00481	1.12415	1558975 at	FAM100A
86	0.00484	1.35716	214682 at	LOC399491
87	0.00493	0.93884	226128 at	NA
88	0.00497	1.10140	216272 x at	SYDE1
89	0.00507	1.14840	213502 x at	LOC91316
90	0.00510	1.12802	1556689 a at	WNT4
91	0.00510	1.25845	212831 at	MEGF9
92	0.00531	1.07698	213804 at	INPP5B
93	0.00541	0.86834	223158 s at	NEK6
94	0.00574	0.85240	240154 at	NA
95	0.00576	1.31384	218812 s at	ORAI2
96	0.00576	1.42638	232311 at	B2M
97	0.00577	0.84905	210250 x at	ADSL
98	0.00580	1.36195	1560390 s at	LOC284440
99	0.00591	0.90175	236328 at	ZNF285A
100	0.00594	0.86716	211537 x at	MAP3K7

7720 genes are significant at the nominal 0.01 level of the univariate test

BV6 sensitive	Parametric p-value	Geometric mean of intensities (class BV6 /class DMSO)	Probe set	Gene symbol
1	0.00001	0.12222	224428 s at	CDCA7
2	0.00001	17.81849	214236 at	CDC27
3	0.00003	0.18729	209330 s at	HNRNPD
4	0.00003	0.19259	213226 at	CCNA2
5	0.00003	0.15880	200627 at	PTGES3
6	0.00003	0.19916	225834 at	GCUD2
7	0.00003	0.18342	241716 at	HSPD1
8	0.00004	0.17412	222848 at	CENPK
9	0.00004	0.21574	205010 at	GNL3L
10	0.00004	0.11197	201202 at	PCNA
11	0.00004	0.16200	204444 at	KIF11
12	0.00005	4.92492	237137 at	SCARNA2
13	0.00005	0.16761	217724 at	SERBP1
14	0.00005	0.22196	200052 s at	ILF2
15	0.00005	0.20647	228334 x at	KIAA1712
16	0.00006	0.19201	208405 s at	CD164
17	0.00006	0.18998	223156 at	MRPS23
18	0.00006	0.20542	212281 s at	TMEM97
19	0.00006	0.17754	234000 s at	PTPLAD1
20	0.00007	0.25591	201555 at	MCM3
21	0.00007	0.23397	202918 s at	MOBKL3
22	0.00007	0.22224	201589 at	SMC1A
23	0.00007	0.22558	200883 at	UQCRC2
24	0.00007	0.20944	221766 s at	FAM46A
25	0.00008	0.23429	201947 s at	CCT2
26	0.00008	0.22866	212433 x at	RPS2
27	0.00008	0.15829	201054 at	HNRNPA0
28	0.00008	0.24636	200751 s at	HNRNPC
29	0.00008	0.17937	211762 s at	KPNA2
30	0.00008	0.26264	213911 s at	H2AFZ
31	0.00008	0.24944	1552329 at	RBBP6
32	0.00008	0.21059	200864 s at	RAB11A
33	0.00008	0.15786	200806 s at	HSPD1
34	0.00008	0.17300	201013 s at	PAICS
35	0.00008	0.13461	223809 at	RGS18
36	0.00008	0.24072	200651 at	GNB2L1
37	0.00009	0.26163	201138 s at	SSB
38	0.00009	0.23396	200046 at	DAD1
39	0.00009	0.27414	212314 at	KIAA0746
40	0.00009	0.25159	209628 at	NXT2
41	0.00009	0.23586	208079 s at	AURKA
42	0.00009	0.27847	224320 s at	MCM8
43	0.00009	0.22873	211991 s at	HLA-DPA1
44	0.00009	0.20788	218663 at	NCAPG
45	0.00009	0.25458	238021 s at	hCG 1815491
46	0.00010	0.23501	212408 at	TOR1AIP1
47	0.00010	0.21747	202892 at	CDC23
48	0.00010	0.27297	225676 s at	WDSOF1
49	0.00010	0.25774	208739 x at	SUMO2
50	0.00010	0.20182	203593 at	CD2AP
51	0.00010	0.24706	200638 s at	YWHAZ
52	0.00010	11.14336	1558048 x at	NA
53	0.00010	0.24682	202527 s at	SMAD4
54	0.00010	0.26225	213320 at	PRMT3
55	0.00010	5.95341	225767 at	LOC284801
56	0.00010	0.27331	215780 s at	SET
57	0.00010	0.19589	203316 s at	SNRPE
58	0.00011	0.25364	219493 at	SHCBP1
59	0.00011	0.19422	212085 at	SLC25A6
60	0.00011	7.81470	244612 at	NA
61	0.00011	0.26790	224914 s at	CIP29
62	0.00011	0.24075	201386 s at	DHX15
63	0.00012	0.27761	229067 at	SRGAP2P1
64	0.00012	0.29540	201051 at	ANP32A
65	0.00012	0.25680	200029 at	RPL19
66	0.00012	0.29719	218238 at	GTPBP4
67	0.00012	0.29933	224973 at	FAM46A
68	0.00012	0.25692	202557 at	HSPA13
69	0.00012	0.15564	202431 s at	MYC
70	0.00012	0.24022	213564 x at	LDHB
71	0.00012	0.25394	244033 at	C14orf145
72	0.00012	0.29189	230416 at	NA
73	0.00013	0.24850	1558943 x at	ZNF765
74	0.00013	5.60502	238876 at	NA
75	0.00013	0.29577	222623 s at	ZNF639
76	0.00013	0.27478	200019 s at	FAU
77	0.00013	0.24227	211939 x at	BTF3
78	0.00013	3.54930	1565579 at	NA
79	0.00013	0.25696	221532 s at	WDR61
80	0.00013	0.29484	213687 s at	RPL35A
81	0.00013	0.18373	202232 s at	EIF3M
82	0.00013	0.25238	208750 s at	ARF1
83	0.00013	0.24980	208821 at	SNRPB
84	0.00013	0.29489	202941 at	NDUFV2
85	0.00013	0.27451	223267 at	RG9MTD1
86	0.00013	0.28738	212623 at	TMEM41B
87	0.00013	0.19364	225716 at	NA
88	0.00014	0.28631	212191 x at	RPL13P12
89	0.00014	0.20151	201930 at	MCM6
90	0.00014	0.20663	200082 s at	RPS7
91	0.00014	0.29140	204192 at	CD37
92	0.00014	0.11201	235060 at	LOC100190986
93	0.00014	0.24475	229305 at	MLF1IP
94	0.00014	0.27517	201030 x at	LDHB
95	0.00014	0.19984	207165 at	HMMR
96	0.00014	0.26700	200650 s at	LDHA
97	0.00014	0.24643	225509 at	SAP30L
98	0.00014	0.24425	208687 x at	HSPA8
99	0.00014	0.27419	224587 at	SUB1
100	0.00015	0.24518	235551 at	WDR4

Supplemental Table 4: Differentially regulated Biocarta pathways among BV6- and DMSO-treated primary AML samples, according to LS/KS permutation testing (Pathway comparison). Light grey script indicated pathways not significant in LS/KS testing, but in GSA test.

Biocarta Pathway	Pathway description	Number of genes	LS p-value	KS p-value	Efron-Tibshirani's GSA test p-value
**pathways specifically deregulated in BV6 sensitive samples**					
h_DNAfragmentPathway	Apoptotic DNA fragmentation and tissue homeostasis	15	0.00003	0.04441	< 0.005
h_smPathway	Spliceosomal Assembly	22	0.00003	0.04504	< 0.005
h_il18Pathway	IL 18 Signaling Pathway	7	0.00136	0.00337	< 0.005
h_rabPathway	Rab GTPases Mark Targets In The Endocytotic Machinery	25	0.00242	0.04861	< 0.005
h_tnfr1Pathway	TNFR1 Signaling Pathway	37	0.0038	0.00918	< 0.005
h_cpsfPathway	Polyadenylation of mRNA	9	0.00931	0.01273	< 0.005
h_eif2Pathway	Regulation of eIF2	18	0.01638	0.01819	< 0.005
h_d4gdiPathway	D4-GDI Signaling Pathway	21	0.02449	0.28193	< 0.005
h_RacCycDPathway	Influence of Ras and Rho proteins on G1 to S Transition	34	0.02801	0.05138	< 0.005
h_prc2Pathway	The PRC2 Complex Sets Long-term Gene Silencing Through Modification of Histone Tails	13	0.03252	0.10773	< 0.005
h_bard1Pathway	BRCA1-dependent Ub-ligase activity	5	0.04497	0.08486	< 0.005
h_caspasePathway	Caspase Cascade in Apoptosis	36	0.04637	0.51603	< 0.005
h_aifPathway	Opposing roles of AIF in Apoptosis and Cell Survival	5	0.29515	0.46769	< 0.005
h_nfkbPathway	NF-kB Signaling Pathway	25	0.42002	0.08742	< 0.005
**pathways specifically deregulated in BV6 resistant samples**					
h_ctlPathway	CTL mediated immune response against target cells	16	0.00614	0.59215	< 0.005
h_ionPathway	Ion Channel and Phorbal Esters Signaling Pathway	7	0.00849	0.02759	< 0.005
h_agpcrPathway	Attenuation of GPCR Signaling	13	0.0099	0.00023	< 0.005
h_chrebpPathway	ChREBP regulation by carbohydrates and cAMP	12	0.01466	0.00003	< 0.005
h_raccPathway	Ion Channels and Their Functional Role in Vascular Endothelium	11	0.015	0.0001	< 0.005
h_il2Pathway	IL 2 signaling pathway	22	0.01677	0.01018	< 0.005
h_pdgfPathway	PDGF Signaling Pathway	33	0.01736	0.00807	< 0.005
h_mhcPathway	Antigen Processing and Presentation	14	0.0193	0.01398	< 0.005
h_gcrPathway	Corticosteroids and cardioprotection	23	0.02473	0.001	< 0.005
h_gsPathway	Activation of cAMP-dependent protein kinase, PKA	14	0.02478	0.00052	< 0.005
h_plcePathway	Phospholipase C-epsilon pathway	15	0.0251	0.00014	< 0.005
h_egfPathway	EGF Signaling Pathway	36	0.02617	0.00662	< 0.005
h_pkcPathway	Activation of PKC through G protein coupled receptor	8	0.03227	0.02533	< 0.005
h_cremPathway	Regulation of Spermatogenesis by CREM	8	0.0343	0.00133	< 0.005
h_il4Pathway	IL 4 signaling pathway	7	0.03687	0.12185	< 0.005
h_ifngPathway	IFN gamma signaling pathway	13	0.04199	0.09419	< 0.005
h_myosinPathway	PKC-catalyzed phosphorylation of inhibitory phosphoprotein of myosin phosphatase	22	0.04362	0.01643	< 0.005
h_cftrPathway	Cystic fibrosis transmembrane conductance regulator (CFTR) and beta 2 adrenergic receptor (b2AR) pathway	12	0.0482	0.00287	< 0.005
h_stressPathway	TNF/Stress Related Signaling	31	0.04909	0.00733	< 0.005
h_mPRPathway	How Progesterone Initiates the Oocyte Maturation	22	0.05098	0.01763	< 0.005
h_tubbyPathway	G-Protein Signaling Through Tubby Proteins	8	0.05217	0.02533	< 0.005
h_bArrestinPathway	?-arrestins in GPCR Desensitization	15	0.05415	0.00401	< 0.005
h_nos1Pathway	Nitric Oxide Signaling Pathway	6	0.10852	0.03352	< 0.005
h_ifnaPathway	IFN alpha signaling pathway	14	0.11202	0.02918	< 0.005
h_ptenPathway	PTEN dependent cell cycle arrest and apoptosis	36	0.17184	0.04242	< 0.005
h_barr-mapkPathway	Role of β-arrestins in the activation and targeting of MAP kinases	18	0.17544	0.03456	< 0.005
h_atrbrcaPathway	Role of BRCA1, BRCA2 and ATR in Cancer Susceptibility	23	0.5561	0.59134	< 0.005
**pathways deregulated in both BV6 sensitive and resistant samples**					
h_setPathway	Granzyme A mediated Apoptosis Pathway	20	0.00001	0.01475	< 0.005
h_glycolysisPathway	Glycolysis Pathway	17	0.00015	0.00047	0.115
h_iresPathway	Internal Ribosome entry pathway	16	0.00016	0.03141	< 0.005
h_eifPathway	Eukaryotic protein translation	25	0.00249	0.00867	< 0.005
h_SARSpathway	SARS Coronavirus Protease	10	0.00338	0.24913	< 0.005
h_ck1Pathway	Regulation of ck1/cdk5 by type 1 glutamate receptors	11	0.01807	0.00065	< 0.005
h_hifPathway	Hypoxia-Inducible Factor in the Cardiovascular System	26	0.03089	0.18056	< 0.005
